# Emotion Recognition Based on Skin Potential Signals with a Portable Wireless Device

**DOI:** 10.3390/s21031018

**Published:** 2021-02-02

**Authors:** Shuhao Chen, Ke Jiang, Haoji Hu, Haoze Kuang, Jianyi Yang, Jikui Luo, Xinhua Chen, Yubo Li

**Affiliations:** 1 College of Information Science and Electronic Engineering, Zhejiang University, Hangzhou 310027, China; 21931039@zju.edu.cn (S.C.); 21960347@zju.edu.cn (K.J.); haoji_hu@zju.edu.cn (H.H.); 11831027@zju.edu.cn (H.K.); yangjy@zju.edu.cn (J.Y.); jack_luo@zju.edu.cn (J.L.); 2 Zhejiang Key Laboratory for Pulsed Power Tanslational Medicine, Hangzhou Ruidi Biotech Ltd., Hangzhou 310000, China; xinhua_chen@zju.edu.cn

**Keywords:** emotion recognition, gradient-boosting decision tree, skin potential, portable device

## Abstract

Emotion recognition is of great importance for artificial intelligence, robots, and medicine etc. Although many techniques have been developed for emotion recognition, with certain successes, they rely heavily on complicated and expensive equipment. Skin potential (SP) has been recognized to be correlated with human emotions for a long time, but has been largely ignored due to the lack of systematic research. In this paper, we propose a single SP-signal-based method for emotion recognition. Firstly, we developed a portable wireless device to measure the SP signal between the middle finger and left wrist. Then, a video induction experiment was designed to stimulate four kinds of typical emotion (happiness, sadness, anger, fear) in 26 subjects. Based on the device and video induction, we obtained a dataset consisting of 397 emotion samples. We extracted 29 features from each of the emotion samples and used eight well-established algorithms to classify the four emotions based on these features. Experimental results show that the gradient-boosting decision tree (GBDT), logistic regression (LR) and random forest (RF) algorithms achieved the highest accuracy of 75%. The obtained accuracy is similar to, or even better than, that of other methods using multiple physiological signals. Our research demonstrates the feasibility of the SP signal’s integration into existing physiological signals for emotion recognition.

## 1. Introduction

Emotion is an important characteristic of human beings, which affects humans’ physical and mental states. Empowering computers and robots to understand human emotions would make human–machine interaction more meaningful and useful for various applications [1]. For instance, in a shopping recommendation system, the computer may make accurate personalized recommendations based on the user’s emotions [2]. Emotion recognition is also important for medical applications, such as the identification of mental problems so that proper medication and preventative measures can be taken.

Many efforts have been made to recognize human emotions. Audiovisual methods are one of the non-contact methods used to catch emotion expressions, i.e., the facial expressions, speech, and gestures used for analysis [3,4,5,6]. However, there are limitations to these approaches, because people can deliberately hide or disguise emotions by controlling their voices and facial expressions. In addition, obtaining audiovisual signals requires the cooperation of subjects; thus, these are difficult to use in most medical applications. 

On the other hand, physiological signals are usually considered to be involuntary signals, and are hence more natural and useful for emotional recognition [7]. Electrocardiography (ECG), electroencephalography (EEG) and galvanic skin response (GSR), etc., are widely used physiological signals, which have been found to be strongly correlated with emotions [8,9,10,11,12,13]. 

In this paper, we study emotion recognition based on the skin potential (SP) signal, which is another physiological signal that has been relatively ignored. SP has been considered to be correlated with the change in emotions since the 1880s [14]. Between 1880 and 1889, Tarchanoff stimulated subjects’ emotions and memories and used a very sensitive galvanometer to measure changes in SP [14]. SP is one method used to record the galvanic skin response (GSR) [15]. However, nowadays, skin conductance (SC), which is another method of recording GSR, has been much more widely used in psychophysiological measurements than SP [16,17]. The reason for this is that the SP response (SPR) is composed of two underlying processes that drive the SP in opposite directions, making the evaluation of SPR amplitudes problematic [17]. Compared with its use in psychophysiological measurement, SP measurements are more frequently used within neurology for assessment of the autonomous nervous system functionality, where the term sympathetic skin response is used [18].

Previous research [17,19,20] revealed that the correlation between SP and SC changes due to different situations, such as sex and type of stimulation received. The SP signal contained unique information compared with SC. Wilcott et al. [21] believed that the complex waveform of the SP signal might contain additional psychological significance. This encourages us to develop a technique to acquire SP signal and build an SP-based emotion recognition system to explore the feasibility of using the SP signal for emotion recognition.

In this paper, a portable device was developed to measure the SP signals. Through experimentation, we found that the SP signals obtained between the middle finger and the left wrist are sensitive to emotion changes. Hence, a video induction experiment was designed to stimulate four typical emotions (happiness, sadness, anger, fear) in 26 subjects and obtain their corresponding SP signals. Based on the device and video induction, we obtained a dataset consisting of 397 emotion samples. We then extracted 29 features from each of the emotion samples and used eight well-established algorithms to classify the four emotions based on these features. Experimental results show that the gradient-boosting decision tree (GBDT), logistic regression (LR) and random forest (RF) algorithms achieved the highest accuracy of 75%. We recommend the GBDT algorithm because it obtains balanced classification errors for the four emotions. Our experiments demonstrate the feasibility of SP signals for emotion recognition. 

The remainder of this paper is given as follows. Section 2 summarizes the related work of emotion recognition based on physiological signals. Section 3 introduces the portable device used to collect the SP signals, the characteristics of the SP signals and factors affecting the acquirement of SP signals. In Section 4, we describe the experimental setup of emotion sample collection. Section 5 introduces data preprocessing, feature extraction and the GBDT algorithm. Section 6 provides the experimental results and Section 7 concludes the paper.

## 2. Related Works

With the widespread application of machine-learning algorithms, many researchers use different emotion-induction methods to collect physiological signals of subjects in different emotional states, and implement algorithms to build emotion recognition models. Kim et al. collected four kinds of physiological signal (ECG, respiration, electromyogram and skin conductance) from three subjects by using music to induce emotions, and combined pseudoinverse linear discriminant analysis (pLDA) and emotion-specific multilevel dichotomous (EMDC) algorithms to recognize four different emotions (joy, anger, sadness, pleasure). The overall recognition accuracy rate reached 69.70% [3]. Wen et al. induced joy and sadness emotions in subjects through movies, and recorded their ECG signals. Fisher projection algorithm was selected to classify these two emotions, and an accuracy of 85% was obtained [22]. Hsu et al. used music induction to combine expert selection and subject selection to induce emotions of joy, tension, sadness and peacefulness. They collected the ECG signals of 61 subjects, and extracted a large number of ECG signal features in the time domain, frequency domain and nonlinear analysis. Finally, the least-squares support vector machine (LS-SVM) algorithm was used to build an emotion recognition model based on these features. The overall accuracy for the four emotions was 61.52% [23]. 

Generally, emotion-recognition methods based on physiological signals rely on the use of complicated and expensive equipment for signal acquisition [3,23,24]. With the progress of modern electronics, wearable/portable devices have gradually been developed to collect physiological signals, with the advantages of wearability/portability, wireless capability, and continuous monitoring without causing difficulties in users’ daily lives [25]. Athavipach et al. [26] discussed a preliminary study to develop a wearable device that is a low-cost, single-channel, dry contact, in-ear EEG suitable for non-intrusive monitoring. The device is able to classify four emotions (happiness, calmness, sadness, and fear) with an accuracy of 53.72%. Lin Shu et al. [27] used videos to induce three target emotions (neutral, happiness, and sadness) and collected the heart rate data from a wearable smart bracelet. The overall accuracy for the three emotions was 84%. Domínguez-Jiménez et al. [28] developed a reliable methodology for emotion recognition using wearable devices to measure heart rate, and SC. Šalkevicius et al. [29] used wearable biofeedback sensors to collect blood volume pressure (BVP), SC, and skin temperature from subjects to classify four anxiety levels (low, mild, moderate, and high), and obtained 86.3% accuracy. AN increasing number of researchers are using wearable/portable devices to collect physiological signals for emotion recognition, which will promote the application of emotion-recognition technology based on physiological signals in people’s daily lives. 

## 3. Characteristics of SP Signals

### 3.1. Design of Portable SP Signal Acquisition Device

**Figure 1:**
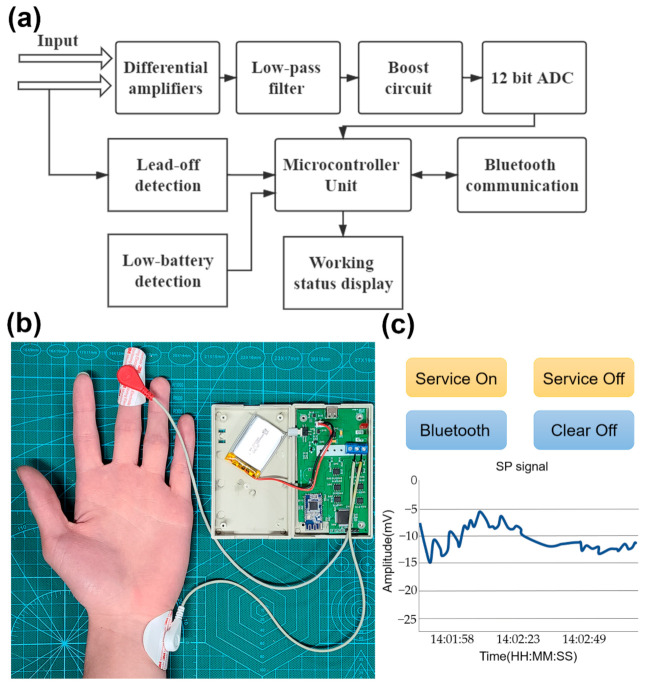
(**a**) Block diagram of the circuit functions of the portable device. (**b**) The portable device for skin potential (SP) signal acquisition. (**c**) The screenshot of the application when it receives signals.

Firstly, we designed a portable wireless device to collect the SP signals. Figure 1a shows the block diagram of the designed circuits. Figure 1b illustrates the portable device. It consists of a small box containing electronics and two electrodes, which are to be attached to the middle finger and left wrist. The device collects SP signals at a sampling rate of 5 Hz, and transmits them to a mobile phone through the Bluetooth wireless communication module for real-time display and storage.

We selected the AD620 chip as the preamplifier, which is a low-cost and high-accuracy instrumentation. It has a large impedance of 10 GΩ and a high common mode rejection ratio (100 dB). Therefore, the resistance of the human body can be neglected, making the measurement of skin potential accurate. A low-pass filter module was used to filter out noise above 10 Hz, so that all collected physiological signals do not face interference from environmental power frequency noise. The boost circuit lifts the output voltage from the last module to a positive value to meet the input demand of ADC. The accuracy of 12-bit ADC is enough to ensure the reliability of the signal after analog-to-digital conversion. Finally, the digital signal is processed in a microcontroller unit and sent to the connected mobile phone through Bluetooth. The device also has the functions of detecting low-battery, lead-off and connection status, and provides timely warnings for abnormalities in the system.

A mobile application is developed for this device. The application connects with the device through Bluetooth and receives the transmitted signal in real-time for display and storage. Figure 1c shows a screenshot of the application when it receives signals. The functionality of Button “Bluetooth” is to switch among all Bluetooth devices and select a specific device to connect with. The button “Clear Off” is used to clear the display of the current signal. Because the mobile phone has more than one application, the “Service On” button ensures that signal recording is not interrupted by other applications. Correspondingly, the “Service Off” button is used to close this functionality.

It is worth mentioning that our signal acquisition method is a passive monitoring method, which does not apply any electrical stimulation to the user; thus, it would not cause any harm to the human body. In addition, the portable device can easily be converted to a wearable device for future application because of its small size of 10 × 6 × 3 cm. Compared with the expensive and complicated bio-amplifiers on the market [30], our device focuses on the acquisition of SP signals. Therefore, our device is cheaper and more convenient to use.

We have conducted a systematic studied on the SP signals generated by multiple parts of the human hands, including elbows, wrists and fingers, and, finally, we found that the SP signals between the fingers and the wrist are more sensitive to emotional responses than those between the elbow and the wrist. Thus, the middle finger and the inner side of the left wrist were selected as the measurement and reference points, as shown in Figure 1b. Please refer to Section 4.1 for details of the comparison experiments.

### 3.2. Analysis of Amplitude-Frequency Characteristics of SP signals

**Figure 2:**
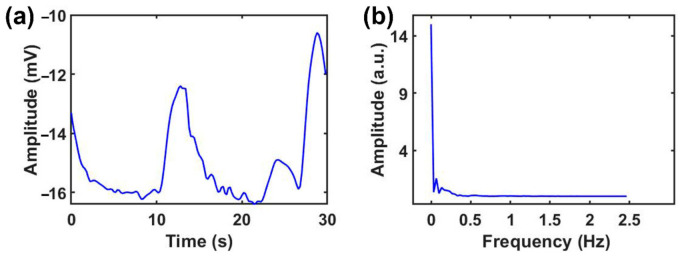
Characteristics the of SP signal. (**a**) The original signal spectrum, and (**b**) frequency spectrum analysis of the signal.

Figure 2a shows a SP signal collected by the device. It can be seen that the amplitude of the SP signal typically ranges from −10 to −17 mV. Figure 2b is the result of unilateral spectrum analysis of the signal after fast Fourier transform (FFT) in Figure 2a. It is obvious that most of the energy of SP signals is accumulated within extremely low frequencies, below 1 Hz. We observed similar amplitude and frequency ranges for all subjects. 

### 3.3. Factors Affecting SP Signals

The purpose of this paper is to study the relationship between the SP signals and emotion states, so it is necessary to exclude other factors which would affect the SP signals. By our experiments, we summarize the factors that may affect the SP signals as follows:

- Rapid changes in temperature;
- Rapid movements of the subject;
- Talking with others;
- After putting on the portable device, the subjects usually cannot calm down quickly, which would make the SP signal unstable.

Therefore, we took the following measures for experiments. First, the experiments were carried out in a controlled temperature scene. Second, we told the subjects not to move or talk deliberately during the experiment. In addition, after putting on the portable device, we waited for 2 min until the SP signal became stable.

## 4. Experiment

### 4.1. Preliminary Experiments

**Figure 3:**
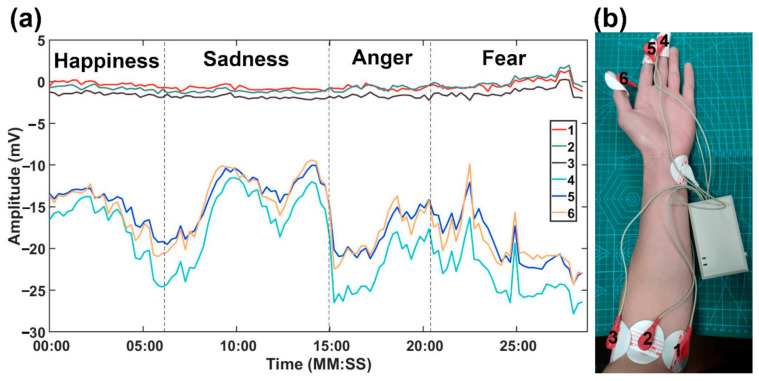
(**a**) SP record diagram of six points from a subject during video viewing. (**b**) The testing points.

To find measurement points that are sensitive to emotions, we conducted the following preliminary experiments. A portable device with six measurement electrodes and one reference electrode was used to collect SP signals. While a subject is watching the video, the six measurement electrodes are placed on three points of the elbow and three fingers, respectively. The reference electrode is placed on the inner side of the left wrist, as shown in Figure 3b. All SP signals are obtained by the potential differences between the measurement points and the reference, that is, the potential of the red point minus the potential of the white point, as shown in Figure 3b.

Figure 3a shows the SP record diagram of subject 2. Lines numbered 1–6 correspond to the six points marked in Figure 3b. Four different emotions are induced in the subject when watching the video (happiness, sadness, anger and fear). The lasting periods of four emotions are divided by the black dashed lines.

It is obvious from Figure 3a that the SP signals at the three points on the fingers are more sensitive to emotion changes than the SP signals at the three points on the elbow. In addition, the three points on the elbow produce similar SP signals, while the three points on the fingers also produce similar SP signals. Thus, we finally chose to place the measurement electrode on the middle finger and the reference electrode on the inner side of the left wrist for the remaining experiments.

### 4.2. Materials and Setup

Experiments were carried out on 26 subjects (seven females and 19 males). Their ages were between 22 and 42 years. All subjects gave their informed consent for inclusion before they participated in the study. The study was conducted in accordance with the Declaration of Helsinki, and the protocol was approved by the Clinical Research Ethics Committee of the First Affiliated Hospital, College of Medicine, Zhejiang University, China (No. 2018YFC0810201).

**Figure 4:**
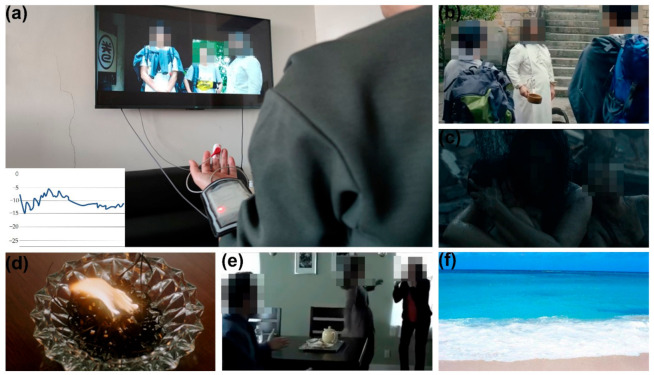
(**a**) The experimental scene and a screenshot of the SP signal on the mobile phone; (**b**) The scene of happiness from the first part of the video (excerpt from the movie NEVER SAY DIE); (**c**) The scene of sadness from the second part of the video (excerpt from the movie Aftershock); (**d**) The scene of anger from the third part of the video (excerpt from the movie God Of Gamblers); (**e**) The scene of fear from the fourth part of the video (excerpt from the movie Insidious); (**f**) The landscape image shown between two adjacent video parts.

The experiment was performed in a laboratory environment with controlled temperature (26 ± 1 °C). The SP signals were collected by the portable device in Section 3.1, and sent to a mobile phone via Bluetooth. A 29 min video was used to stimulate the emotions of the subjects. Figure 4a shows the experimental scene. The subject is sitting on the sofa and watching the video, while the portable device is collecting the SP signals of the subject. The experimenter can observe the subject’s signals in real time on a mobile phone, as shown in Figure 4a.

The video contained four video parts that aimed to stimulate emotions of happiness, sadness, anger and fear, respectively, with the scenes shown in Figure 4b–e. There was a two-minute interval between two adjacent video parts. During this interval, a relaxing landscape image (Figure 4f) and soft music were displayed to cause the subjects to calm down before entering into another emotion state. 

### 4.3. Experimental Protocol

During the experiment, the subject was required to answer a questionnaire, with the following three questions, for each part of the video:

1. What is the emotion aroused after you watch this video part? Please choose one from the four emotions—happiness, sadness, anger and fear. If you think the emotion aroused is not in the above four categories, please choose “others”;
2. Please quantitatively score the degree of emotion aroused by this video part. The score is from 1 to 5, where 1 represents the weakest degree and 5 represents the strongest degree;
3. Please select the exact time periods that arouse this emotion and the corresponding arouse degree for each time period. Each time period is represented as “from MM:SS (the start time) to MM:SS (the end time)”. The score of degree is also from 1 to 5, where 1 represents the weakest degree and 5 represents the strongest degree.

Questions 1 and 2 were to be completed by the subject within the two-minute interval between each video. After the subject watched the whole video, the experimenter would help the subject answer Question 3 by dragging the progress bar to replay the video. During the replay, the experimenter should confirm the time periods and degree of emotion with the subject.

**Figure 5:**
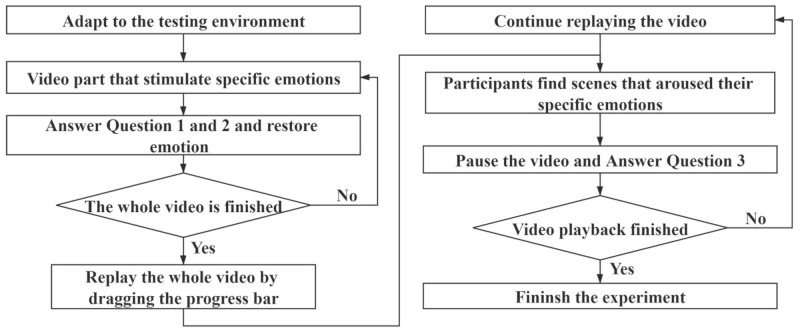
Block diagram of experimental procedures.

Figure 5 shows the block diagram of the experimental procedures. The specific experimental procedures are as follows:

1. Put the electrodes on the middle finger and left wrist of the subject. Turn on the device. Wait for 2 min until the SP signal becomes stable;
2. Play the first part of the video and record the SP signal of the subject on the experimenter’s mobile phone;
3. During the two-minute interval after the video is played, the experimenter asks the subject to complete Questions 1 and 2;
4. Recursively go to Step 2 and 3 to play the remaining three videos, and obtain the SP signals and answers to Questions 1 and 2;
5. After the whole video has been played, take off the subject’s portable device. The experimenter helps the subject answer Question 3 by dragging the progress bar to replay the video and confirms the time periods and degree of emotion with the subject;
6. After the experiment is finished, the experimenter needs to request that the subject sit for several minutes to calm down before they walk out of the laboratory.

The experimental results of Question 1 indicate that most subjects felt the emotion assigned to each video part, except for four subjects, who misclassified the anger emotion as “others”. For Question 2, the average degree scores for four emotions are 3.46, 3.62, 3.15 and 4.08, respectively, indicating that the emotions aroused by the video parts were strong enough for recognition.

## 5. Methodology

**Figure 6:**
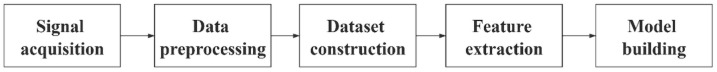
Block diagram of the construction procedure of SP-based emotion recognition model.

Figure 6 shows the block diagram of the SP-based emotion recognition system, which consists of signal acquisition, data preprocessing, dataset construction, feature extraction and model building. 

### 5.1. Data Preprocessing and Dataset Construction

Firstly, the SP signals for each subject are normalized by the following equation

(1) $$x\_norm=\left(x-x\_min\right)/\left(x\_max-x\_min\right)$$

where x_min and x_max represent the minimum and maximum values of the original signal *x*.

**Figure 7:**
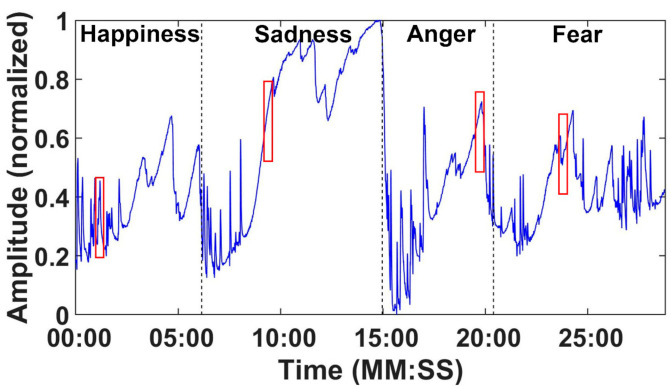
The whole SP recording from subject 5 during video viewing after data preprocessing.

Figure 7 shows one example of the SP record diagram of subject 5 after data preprocessing. The black dashed lines represent the end time of each video section. For our recognition system, we only selected the time periods which aroused strong emotions as emotion samples for training and testing. In our experiment, all emotion samples lasted for 30 s. The selection principles are listed as follows:

1. Select the time period with a degree score greater than 3;
2. If the selected time period is less than 30 s, then expand the time period to 30 s by filling the gap equally before and after this time period;
3. If the selected time period is more than 30 s, then expand the time period to a multiple of 30 s and equally divide it into several 30-s time periods.

In much of the related research, emotion samples are segmented by fixed lengths. For example, Kim et al. [7] and Hsu et al. [23] selected 50 s and 1 min as the lengths for emotion samples, respectively. According to the answers of Question 3, the lengths of most time periods were within 30 s. Hence, we set 30 s as the length of emotion samples. Each emotion sample is represented as a 150-dimensional vector, because the sampling rate is 5 Hz and its length is 30 s. The red rectangles of Figure 7 show four examples of the extracted emotion samples.

**Figure 8:**
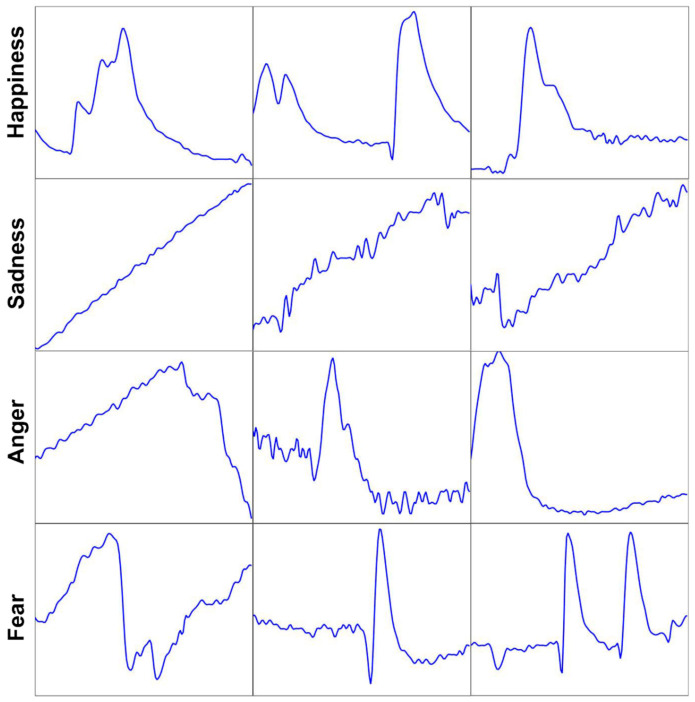
Twelve emotion samples of happiness, sadness, anger and fear.

Figure 8 shows 12 emotion samples of the four emotion classes (three samples for each class). It can be seen that samples of each emotion class have intrinsic characteristics. For example, the emotion samples of happiness and fear rise and fall very rapidly, but the changes in sadness and anger are relatively steady. These intrinsic characteristics are foundations for the design of our emotion recognition algorithm.

### 5.2. Feature Extraction

Following previous research into physiological signal analysis [23,31,32,33,34], we extracted 29 features from each emotion sample, which include 15 time-domain features, 13 frequency-domain features and 1 nonlinear feature. Table 1 lists details of these extracted features.

#### 5.2.1. Time-Domain Features

The time-domain features include the first quartile (q1), median value (median), the third quartile (q3), mean value (mean), standard deviation (std), variance (var) and root mean square (rms) of the original emotion samples. In addition, the maximum ratio (max_ratio) and minimum ratio (min_ratio) of the original emotion samples are also calculated by Equations (2) and (3)

(2) $$min\_ratio=\left(x\_min\right)/\left(len\_x\right)$$

(3) $$max\_ratio=\left(x\_max\right)/\left(len\_x\right)$$

where len_x represents the data length of the signal. In addition, we calculated the first-order differentiation and the second-order differentiation from the emotion sample, and their mean (diff1_mean and diff2_mean), median (diff1_median and diff2_median) and standard deviation (diff1_std and diff2_std) were also obtained as the time domain features.

#### 5.2.2. Frequency Domain Features

In order to extract the frequency domain features of the SP signals, we first used fast Fourier transform (FFT) on the emotion samples to extract the unilateral spectrum. Subsequently, the unilateral spectrum was calculated to obtain its mean (mean_f), median (median_f), variance (var_f), standard deviation (std_f), root mean square (rms_f), maximum ratio (max_ratio_f) and minimum ratio (min_ratio_f) as the frequency domain features. Then, the first- and second-order differentiations were calculated from the unilateral spectrum. We further obtained their means (diff1_mean_f and diff2_mean_f), medians (diff1_median_f and diff2_median_f) and standard deviations (diff1_std_f and diff2_std_f) as the frequency domain features.

#### 5.2.3. Nonlinear Features

The only non-linear feature extracted in our experiments was the mean crossing rate of the signal (mcr), which refers to the number of times the signal crosses the average value. This measures the vibration level of the signal.

### 5.3. Classifier Construction

We use the gradient-boosting decision tree (GBDT) algorithm to classify the four emotions based on the extracted 29 features. The idea of GBDT was first proposed by Friedman [35]. It is a powerful ensemble machine-learning algorithm that produces a prediction model in the form of an ensemble of weak learners, typically decision trees such as classification and regression trees [36]. Compared with traditional classifiers, GBDT can produce competitive, robust and interpretable procedures for both classification and regression, which are especially appropriate for mining unclean data [35]. Hence, GBDT has been widely used in radar target recognition [36], intrusion detection systems [37], hand gesture recognition [38] and indoor localization [39].

For our emotion recognition task, take N training samples ${\left\{X_{i},Y_{i}\right\}}^{N}$, where $X_{i}$ is the i-th emotion sample with 150 dimensions. $Y_{i}$ = ($y_{i1}$, $y_{i2}$, … $y_{iK}$) is the one-hot ground-truth label for $X_{i}$, and K represents the number of classes (K=4 in our experiment, because we need to classify four emotions). The general training procedures are given as follows.

Initializing the model

(4) $$F_{k,0}\left(X\right)=0\;\;\;,k=1,2,\ldots ,K$$

where $F_{k,0}\left(X\right)$ represents the initial decision tree of the k-th class.

(1) For iteration m = 1:M:

(a) Calculating the probability that the training sample belongs to each class by Equation (5)

(5) $$P_{k,m-1}\left(X\right)=exp\left(F_{k,m-1}\left(X\right)\right)/\sum_{l=1}^{K}exp\left(F_{l,m-1}\left(X\right)\right),\;k=1,2,\ldots ,K$$

where m refers to the m-th iteration;

(b) Calculating the approximation of residual for each class and each sample

(6) $${\tilde{r}}_{ik}=y_{ik}-P_{k,m-1}\left(X_{i}\right),\;i=1,2,\ldots ,\;N,\;k=1,2,\ldots ,K$$

(c) Fitting decision trees of each class to the approximation of residual

(7) $${\left\{R_{jkm}\right\}}_{j=1}^{J}=J-\mathrm{leaf}\;\mathrm{node}\;\mathrm{tree}\left({\left\{{\tilde{r}}_{ik},X_{i}\right\}}_{1}^{N}\right)$$

where j  refers to the j-th leaf, k refers to the k-th decision tree, and m refers to the m-th iteration. $R_{jkm}\;$ is the specific leaf space;

(d) The new step-size of model can be computed with

(8) $$\beta_{jkm}=\left(K-1\right)\sum_{X_{i}\in R_{jkm}}{\tilde{r}}_{ik}/(K\sum_{X_{i}\in R_{jkm}}\left|{\tilde{r}}_{ik}\right|\left(1-\left|{\tilde{r}}_{ik}\right|\right))$$

(e) Updating model with Equation (9)

(9) $$F_{k,m}\left(X\right)=\;F_{k,m-1}\left(X\right)+\sum_{j=1}^{J}\nu \cdot \beta_{jkm}I\left(X_{i}\in R_{jkm}\right),0<\nu \leq 1$$

where $I\left(\cdot \right)$ is the indicator function, which equals 1 if its argument is true and 0 otherwise. ν is the learning rate.

The testing procedure used Equation (5) to calculate the probability that a testing sample X belonged to each class. The class with the maximum probability is the one predicted by the model.

## 6. Experimental Results and Discussions

We collected 397 emotion samples from 26 subjects, which included 85 happiness, 135 sadness, 42 anger and 135 fear samples. We split the emotion samples into the train and test sets, as shown in Table 2. Generally, we used the samples of 19 subjects for training and the remaining seven subjects for testing. In this way, we used data from different subjects to train and test the classification models, avoiding the data dependency problem.

We used eight algorithms based on feature selection to classify the datasets, which included K-nearest neighbor (KNN), neural network (NN), linear discriminant analysis (LDA) [40], logistic regression (LR) [41], random forest (RF) [27,41,42], decision tree (DT) [42,43], support vector machines (SVM) [23] and gradient boost decision tree (GBDT). All algorithms were implemented with Python sklearn library. The hyperparameter settings for the above algorithms are listed in Table 3.

In the process of building the recognition model, we set a five-fold cross-validation of the train set to evaluate the performance of different parameters, and to finally determine a better combination of parameters to build the recognition model. 

**Figure 9:**
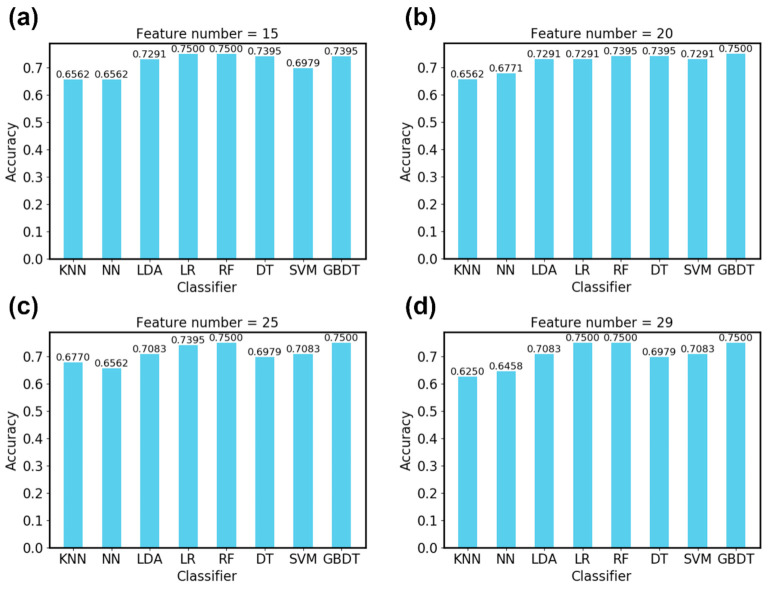
The classification accuracy on the test set of eight classifiers when (**a**) 15 features are selected; (**b**) 20 features are selected; (**c**) 25 features are selected; (**d**) 29 features are selected.

Figure 9 shows the classification accuracy of each algorithm on the test set when a different number of features is selected. Here, the “SelectKBest” function of the sklearn library was used for feature selection. The number of features was set to 15, 20, 25 and 29 (all features), respectively. It can be seen that all algorithms obtain an accuracy greater than 65%, which proves the feasibility of using SP signals for emotion recognition.

Figure 9 also shows that LR, RF and GBDT achieved the same highest accuracy 75% when all 29 features were selected. To further compare their performance, we list the accuracy of each emotion for these three algorithms in Table 4. It can be seen that the accuracy of GBDT is more balanced. It obtained the highest accuracy for the anger emotion, which has the fewest samples of the four emotions. Thus, we chose the GBDT algorithm for further experiments.

**Figure 10:**
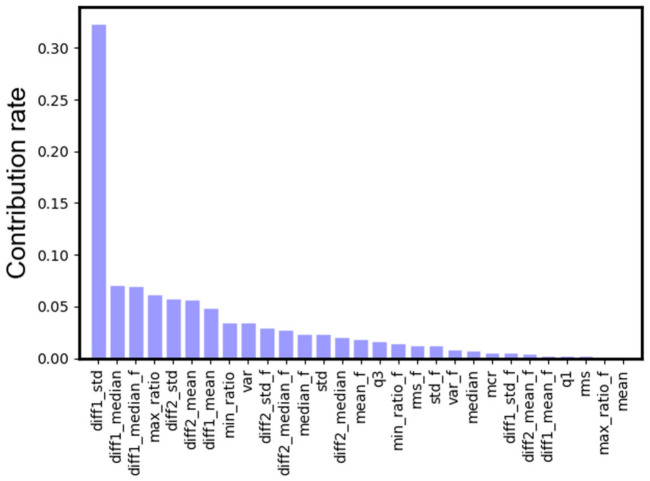
The contribution rates of all 29 features.

In order to understand which feature of SP signal has a stronger correlation with emotion, we plot the contribution rate of each feature in Figure 10. The contribution rate is proportional to the frequency that this feature is selected by the decision trees of GBDT [35]. We find that the standard deviation of the first-order differential (diff1_std) had the largest contribution, followed by the median of the first-order differential (diff1_median). From Figure 8, we can see that the fluctuation in SP signals carries abundant emotion information, so it is not surprising that features related to the degree of fluctuation, such as diff1_std and diff1_median, have a greater impact on the recognition results. 

Table 5 shows the confusion matrix of GBDT on the test set. The accuracies for happiness, sadness, anger and fear are 61.11%, 89.28%, 18.18% and 87.17%, respectively. The accuracy of sadness and fear is relatively high. The accuracy of anger is relatively low, probably because, intuitively, anger is difficult arouse through watching videos. In our experiments, we only collected 42 anger samples, which is the least of the four emotions. This means that the 26 subjects produced degree scores greater than 3 only 42 times. The average degree score for this emotion was 3.15, which indicates that the subjects were also not very confident with their aroused anger emotion.

Another observation is that, in many cases, anger is misclassified as sadness (54.54%). This may indicate that the SP signal collected when the subjects feel angry is similar to the SP signal collected when they feel sad. However, sadness has more emotion samples, so the GBDT algorithm tends to misclassify anger as sadness. Generally, the total recognition rate is 75%, indicating that the SP signals have a discriminative ability for these emotions.

Table 6 shows the accuracy of each subject in the test set. The highest and lowest accuracy was 93.57% and 61.11%, respectively. According to Table 5, GBDT is more accurate in classifying sadness and fear, so, generally, the accuracy is higher for subjects with a higher proportion of sadness and fear emotion samples. 

We further compare the performance of the SP signal with other physiological signals for emotion recognition. Table 7 lists the classification performance of the proposed method, together with other existing methods in the literature. For each line, we listed the signal types, number of subjects, emotions to be recognized, induction methods, classification algorithms and accuracies. However, the accuracy is influenced by various factors, such as signal type, emotions to be recognized, sample distributions and induction methods. The results show that the performance of the proposed methods using only the SP signal is similar to, or even better than, that of other methods using multiple physiological signals. For example, Rainville et al. [44] used the PCA+Heuristic-decision-tree algorithm to process the electrocardiogram and respiration signals and obtained 65.30% accuracy for four emotions, compared with the 75% accuracy of our proposed method for the same emotions. Moreover, our device for obtaining SP signals is simple and portable, compared with complicated and expensive measurement systems [3,23], which is another advantage of the proposed method.

## 7. Conclusions and Future Work

In this paper, the extremely low-frequency SP signal between the middle finger and left wrist was found to be strongly correlated with emotions. A portable wireless device was developed to measure the SP signals for emotion recognition. We extracted 29 features from each of the emotion samples collected in our video induction experiment. Eight classification algorithms were trained to classify four emotions (happiness, sadness, anger and fear) based on these features. Experimental results show that all algorithms obtain an accuracy greater than 65%, and three algorithms (LR, RF and GBDT) achieved the highest accuracy of 75% on the test set. The accuracy of GBDT is more balanced for the four emotions, which is our recommended algorithm. 

The single SP-signal-based emotion recognition method is convenient, simple and obtains a similar or better accuracy than existing complicated and expensive systems. Thus, the SP signal could feasibly be integrated into the existing emotion recognition system based on physiological signals. 

For our future work, we will collect a large number of emotion samples from more subjects and build a more reliable emotion recognition model. In addition, the portable SP-signal-based emotion recognition system could be used in outdoor scenes to obtain more natural emotions. 

## Figures and Tables

**Table 1:** 29 Features Extracted in This Study.

Domain	Feature name
Time-Domain	q1, q3, median, mean, std, var, rmsmin_ratio, max_ratiodiff1_mean, diff1_median, diff1_stddiff2_mean, diff2_median, diff2_std
Frequency-Domain	mean_f, median_f, std_f, var_f, rms_fmin_ratio_f, max_ratio_fdiff1_mean_f, diff1_median_f, diff1_std_fdiff2_mean_f, diff2_median_f, diff2_std_f
Nonlinear	mcr

**Table 2:** Statistics of the Dataset.

	Number of Subjects	Happiness	Sadness	Anger	Fear
Train Set	19	67	107	31	96
Test Set	7	18	28	11	39
Total	26	85	135	42	135

**Table 3:** Hyperparameter Settings of Classification Algorithms (the settings of other parameters follow the default settings of sklearn if not listed).

Classifier	Parameter	Parameter Explanation
K-nearest neighbor (KNN)	leaf_size = 50	leaf_size: leaf node size
neural network (NN)	hidden_layer_sizes = (50,100,100,50,40)solver = “lbfgs”, alpha = 1 × 10^−^^5^	hidden_layer_sizes: the structure of the hidden layersolver: selection of weight-optimization methodalpha: regularization parameter
linear discriminant analysis (LDA)	solver = “svd”, shrinkage = None	solver: choice of solution algorithmshrinkage: whether to use parameter shrink
logistic regression (LR)	penalty = “l2”, solver = “newton-cg”,multi_class = “multinomial”	penalty: regularization selection parametersolver: choice of loss functionmulti_class: choice of classification
random forest (RF)	n_estimators = 1000	n_estimators: the number of trees in the forest
decision tree (DT)	criterion = “gini”, max_depth = 2, splitter = “best”	criterion: the function used to calculate the impurity of the treemax_depth: the maximum depth of the decision treesplitter: the strategy used to choose the split at each node
support vector machines (SVM)	C = 17, gamma = 0.001, kernel = “rbf”	C: penalty coefficientkernel: the choice of kernel functiongamma: the parameter of kernel function “rbf”
gradient boost decision tree (GBDT)	n_estimators = 100, max_depth = 3, min_samples_split = 2, min_samples_leaf = 1, learning_rate = 0.1	n_estimators: maximum number of iterations of the weak learnermax_depth: the maximum depth of the decision treemin_samples_split: parameters that restrict the conditions of subtree divisionmin_samples_leaf: parameters that limit the minimum number of samples of child nodeslearning rate: weight reduction coefficient of each weak learner

**Table 4:** Accuracy of Every Emotion with LR, RF and GBDT Algorithms.

Algorithms	Happiness	Sadness	Anger	Fear
LR	50.00%	92.86%	9.09%	92.31%
RF	66.67%	89.28%	9.09%	87.18%
GBDT	61.11%	89.28%	18.18%	87.18%

**Table 5:** Confusion Matrix on the Test Set.

	Predicted	Happiness	Sadness	Anger	Fear
True					
Happiness		11(61.11%) *	3(16.66%)	2(11.11%)	2 (11.11%)
Sadness		1(3.57%)	25(89.28%) *	1(3.57%)	1(3.57%)
Anger		2(18.18%)	6(54.54%)	2(18.18%) *	1(5.56%)
Fear		4(10.26%)	1(2.56%)	0(0.00%)	34(87.17%) *

The * indicates the accuracy of each emotion.

**Table 6:** The Accuracy of Each Subject in the Test Set.

Subjects	The Number of Emotion Samples				Accuracy
	Happiness	Sadness	Anger	Fear	
S1	6	4	0	1	81.81%
S2	1	6	1	8	93.75%
S3	0	2	2	8	83.33%
S4	2	4	2	6	64.28%
S5	2	5	1	6	71.43%
S6	4	5	4	5	61.11%
S7	3	2	1	5	63.63%
Sum	18	28	11	39	75.00%

**Table 7:** Classification Performance Comparisons of Proposed Emotion Recognition Methods with Some Existing Methods for Multi-Emotion Recognition.

Author	Signals	No. of Subjects	Emotions	Induction Method	Classification Algorithm	Accuracy
Proposed method	Skin potential	26	Happiness, Sadness, Anger, Fear	Videos	GBDT	75% (4 emotions)
Kim et al. [7]	Electrocardiogram, Skin temperature, Electrodermal activity	50	Sad, Anger, Stress, Surprise	Multimodal	SVM	78.4% (3 emotions)61.8% (4 emotions)
Rainville et al. [44]	Electrocardiogram, Respiration	43	Fear, Anger, Sadness Happiness	Recall their personal emotional episode	PCA+Heuristic decision tree	65.3% (4 emotions)
Gu et al. [45]	ElectrocardiogramBlood volumepulse, Skinconductivity, Electromyogram, Respiration rate	28	Positive and High arousal, Negative and High arousal Positive and Low arousal, Negative and Low arousal	Pictures (IAPS)	K-nearest neighbor	50.3% (4 dimensions)
Wen et al. [21]	Electrocardiogram	154	Joy, Sadness	Movies	Fisher projection	85% (2 emotions)
Hsu et al. [23]	Electrocardiogram	61	Joy, Tension, Sadness, Peacefulness	Music	LS-SVM	61.52% (4 emotions)
Kim et al. [3]	Electrocardiogram, Respiration, Electromyogram, Skin conductivity	3	Joy, Anger, Sadness, Pleasure	Music	pLDA+EMDC	69.7% (4 emotions)
Rigas et al. [46]	Electrocardiogram, Respiration, Electromyogram, Galvanic skin response	9	Happiness, Disgust, Fear	Pictures (IAPS)	K-nearestneighbor	62.7% (3 emotions)
Wen et al. [47]	Fingertip blood oxygen saturation, Galvanic skin response, Heart rate	101	Amusement, Anger, Grief, Fear, Baseline	Videos	Random forests	74% (5 emotions)
Lin Shu et al. [27]	Heart rate	25	Happiness, Sadness, Neutral	Videos	Decision tree	84% (3 emotions)

## Data Availability

The data presented in this study are available on request from the corresponding author.
